# Beyond the storms: Exploring predictors of posttraumatic stress and posttraumatic growth among survivors of hurricanes Irma and Maria in Puerto Rico

**DOI:** 10.1016/j.joclim.2024.100365

**Published:** 2024-11-29

**Authors:** Marie-Claire Meadows, Noelle Serino, Dahianira M. Camacho-Monclova, Kaumudi Joshipura, Sarah R. Lowe

**Affiliations:** a Division of Environmental Health Sciences, University of Minnesota School of Public Health, Minneapolis, MN, USA; b Department of Social and Behavioral Sciences, Yale School of Public Health, New Haven, CT, USA; c Center for Clinical Research and Health Promotion, University of Puerto Rico Medical Sciences Campus, San Juan, Puerto Rico, USA; d School of Public Health, Ahmedabad University, Gujarat, India; e Department of Epidemiology, Harvard T.H. Chan School of Public Health, Boston, MA, USA

**Keywords:** Posttraumatic growth, Posttraumatic stress, Hurricanes, Puerto Rico, Disasters

## Abstract

**Background:**

Within disaster-affected communities, residents’ exposures and post-disaster mental health outcomes can vary widely. Yet, few studies have explored the relationship between such diverse disaster-related exposures and posttraumatic growth (PTG) in a Puerto Rican context.

**Methods:**

To address this gap, we used data from the Preparedness to Reduce Exposures and Diseases Post-hurricanes and Augment Resilience (PREPARE) study, a cohort of mainly Hispanic Puerto Ricans who experienced Hurricanes Maria and Irma in 2017. This analysis focused on 484 individuals who completed structured interviews 20 to 34 months after the hurricanes. We evaluated the associations between five different disaster exposures (e.g., financial, home damage, personal health, and familial health), posttraumatic stress (PTS), and PTG and its five domains (personal strength, new possibilities, improved relationships, spiritual growth, and appreciation of life), controlling for demographic, geographic, and social factors.

**Results:**

In multivariable models, higher total disaster score was associated with higher levels of both PTS and PTG (2.91 and 3.87, respectively). Personal health impacts were consistently associated with higher levels on all PTG subscales, ranging from 0.89 to 1.94, which was not the case for other exposures. Specifically, home damage was associated with higher levels on all PTG subscales except spiritual growth, and financial and familial health impacts were associated with greater identification with new possibilities and appreciation of life only.

**Conclusion:**

These findings provide novel evidence that different disaster-related exposures have distinct associations with the different PTG domains in Puerto Rico. These findings can inform future efforts to address post-disaster mental health ailments by bolstering different aspects of PTG.

## 1. Introduction

The frequency and effects of weather-related disasters (e.g., hurricanes, floods), including material, financial, and environmental losses, along with adverse impacts on health and well-being, continue to escalate amidst the intensification of climate change, fueled by the entrance of additional heat and water into the atmosphere and rising global surface temperatures [1,2]. Alongside the rising occurrences and intensity of weather-related disaster events are their corresponding effects on vulnerable communities. Low-income individuals, those with chronic conditions, and displaced populations are among those at highest risk for adverse effects of weather-related disasters as a product of their limited access to assets critical to disaster recovery and overall health [3].

A pair of recent weather-related disasters that exemplifies the disproportionate impact of these events on vulnerable communities are Hurricanes Maria and Irma, which struck Puerto Rico in September of 2017. Puerto Rico, a United States territory in the Caribbean, is particularly susceptible to hurricanes due to its geographic location. Coupled with disparate rates of chronic disease (e.g., diabetes, kidney disease) and poverty relative to other localities in the United States, Puerto Rico is at a disadvantage when enduring and recovering from weather-related disasters, such as those that gained international attention in 2017 [4–6]. Category 5 Hurricane Irma and Category 4 Maria both hit the island within a two-week period, with Maria causing over $90 billion in damage alone [7]. With both disasters amassing extensive infrastructural damage, flooding, prolonged power loss, displacement, and a death toll of nearly 3000, recovery was expected to be labor-intensive and costly [8–11]. Six years later, recovery is still underway, partially due to a lack of access to Federal Emergency Management Agency (FEMA) public assistance funds that are essential to the island’s recovery [12].

Hurricanes Irma and Maria had a profound impact on Puerto Ricans. This included increases in diagnoses of posttraumatic stress disorder (PTSD), major depression, and other anxiety disorders, which have been identified as outcomes associated with weather-related disaster exposure, both through the onset of new symptomatology and the escalation of pre-existing mental illness [13–15]. The relationship between disaster survivorship and these adverse mental health outcomes must be considered within the Puerto Rican context, including the island’s fragile health care system and shortage of mental health specialists, against the backdrop of a recession marked by unemployment and familial separation [16,17]. Coinciding with a stark increase in reported suicides and other mental health symptomatology, it will be critical to explore strategies that support and enhance the behavioral health-related needs of Puerto Ricans moving forward [18].

Initial studies after the hurricanes among Puerto Rican residents have provided critical insight into the state of mental health in the region. A study conducted six months after the disasters in Punta Santiago, a town located on the southeastern coast of Puerto Rico, found that >66 % of survey respondents reported increases in scores for at least one of three mental disorders: major depression, generalized anxiety, and PTSD [19]. Other studies have reported similar findings in the longer-term aftermath of the hurricanes, including increases in self-reported diagnoses of PTSD and depression - outcomes that were associated with elevated exposure to hurricane-related stressors such as personal and/or familial harm or loss, compromised access to resources, unsafe living conditions, and home damage [20–22].

While there is a demonstrable link between disaster survivorship and poor mental health outcomes, research has also demonstrated that disasters can promote posttraumatic growth (PTG). According to Tedeschi and Calhoun (1996), PTG is defined by five overarching domains – improvements in interpersonal relationships, a sense of new possibilities, increased personal strength, positive spiritual change, and a greater appreciation for life – that arise as a result of psychological struggle in the aftermath of a potentially traumatic life event [23]. Extant research exploring the relationship between PTG and mental health conditions have revealed that PTG is correlated with PTSD symptoms [24]. A handful of studies have identified predictors of higher PTG among disaster survivors, including female sex, lower income, higher posttraumatic stress (PTS) scores, and psychosocial constructs, including a greater sense of purpose, religiosity, and perceived social support [25–27].

Within this context, researchers have explored unique and shared predictors of PTS and PTG post-disaster. Prior research has found that these are related constructs, but distinct from each other. Some studies have attempted to shed light on distinctions by examining unique and shared predictors of PTS and PTG. For example, a study of low-income women who survived Hurricane Katrina identified that demographic characteristics (i.e., older age, non-Hispanic Black identity) and more disaster-related stressors were associated with both PTS and PTG, whereas greater purpose in life and religious coping were uniquely associated with PTG [28,29]. Notably, this work has not differentiated between different domains of PTG. The domains of PTG often present in a highly individualized manner; one may experience a single domain while another may exhibit multiple domains simultaneously, thereby warranting a more nuanced review of how PTG is experienced in a post-disaster context. This limitation is significant given that both PTG theory and some empirical findings indicate that different types of potentially traumatic events are likely to elicit different PTG domains [30–32]. For example, studies of PTG among combat veterans have found elevated identification with alterations in interpersonal relationships, new possibilities, and spiritual change whereas those living with chronic, life-threatening health conditions such as cancer align more with changes in appreciation of life and personal strength [33–36]. An additional limitation is that researchers have generally not examined whether different disaster-related exposures are uniquely associated with PTS, PTG, and PTG domains. Such findings could also yield insights into the processes leading to different patterns of post-disaster PTS and PTG, ultimately helping providers promote positive outcomes among survivors [37].

To our knowledge, there have been no studies that concurrently examined PTS and PTG after Hurricanes Irma and Maria, which is notable given their concurrent and extreme nature, coupled with the unique cultural context [38,39]. Against the backdrop of limited research on specific, disaster-related exposures and their association with PTS, PTG, and PTG domains, work in this area could provide crucial insight into how varied disaster-related exposures might facilitate positive mental health outcomes and how these pathways can be leveraged in the aftermath of future climate disasters.

The present study therefore aimed to (1) examine the levels and correlations between PTS and PTG post-Hurricanes Irma and Maria among high-risk adults living in Puerto Rico and (2) identify unique and shared predictors of PTS and PTG in this context.

## 2. Materials and methods

### 2.1. Participants and procedures

The current study drew upon data from the Preparedness to Reduce Exposures and Diseases Post-hurricanes and Augment Resilience (PREPARE) study [40]. PREPARE included participants from: (1) a pre-existing cohort, the San Juan Overweight Adults Longitudinal Study (SOALS) [41]; and (2) from federally qualified health centers, Corporación de Servicios de Salud y Medicina Avanzada (COSSMA), which have been providing outpatient and preventative services for individuals with chronic diseases. SOALS recruited 1,206 overweight/-obese Hispanic adults aged 40 to 65 years, free of major health conditions, between 2011 and 2013, 1028 of whom completed a follow-up survey between 2014 and 2016. Of the 869 that were deemed eligible for participation in PREPARE, 377 resided in San Juan, 281 in the San Juan Metropolitan area, and 211 outside San Juan. PREPARE aimed to include 125 participants from each location. A total of 364 SOALS participants provided post-hurricane data that were included in this study. Due to PREPARE’s initial key interests in diabetes management and incidence, COSSMA participants were considered for inclusion if they were diagnosed with diabetes prior to the hurricanes, attended a medical appointment no earlier than one year before the hurricanes and another visit within the following year; and had pre- and post-disaster data in their health records on HbA1c, height, and weight measurements. Using COSSMA electronic health records, 2,306 patients were identified that qualified for the study. After removing participants with missing information, 416 participants were found eligible for the study, with 125 electing to participate, meeting the targeted sample size goal.

Data collection was conducted between May 2019 and July 2020, 20–34 months post-hurricanes. Eligible and willing participants were scheduled for in-person visits to complete a survey administered in Spanish, have blood drawn, blood pressure measured, and anthropometric measures and hair collected. Following a series of earthquakes in January 2020 and COVID-19 lockdown, 71 interviews with COSSMA patients were conducted over the phone, and 11 SOALS participants were excluded due to in-person restrictions. A total of 489 participants were included in PREPARE. Five participants (1 %) were dropped due to missing data, yielding an analytic sample of 484. This study was approved by the Institutional Review Board at the University of Puerto Rico.

### 2.1. Measures

#### 2.1.1. Posttraumatic stress

PTS was assessed using the Posttraumatic Checklist for DSM-5 (PCL-5), a 20-item measure of DSM-5 PTSD symptom clusters: intrusion (five items, e.g., repeated, disturbing, and unwanted memories of the stressful experience), avoidance (two items, e.g., trouble remembering important parts of the stressful experience), negative alterations in cognition and mood (seven items, e.g., feeling distant or cut off from other people), and alterations in arousal and reactivity (six items, e.g., irritable behavior, angry outbursts, or acting aggressively) [42]. Participants reported how much they were bothered by each symptom in the past month related to their experiences with the hurricanes, from 0 (not at all) to 4 (extremely). Full scale scores were computed as the sum of responses to all items, ranging from 0 to 80. Cronbach’s alpha was 0.91. The PCL-5 has both been validated in Spanish and utilized in a Puerto Rican context [43–46]. For the purposes of our study, the PCL-5 was administered to participants in Spanish.

#### 2.1.2. Posttraumatic growth

PTG was assessed by the Posttraumatic Growth Inventory (PTGI), a 21-item measure assessing the five PTG domains: personal strength (four items, e.g., “I have a greater feeling of self-reliance”), new possibilities (five items, e.g., “I have developed new interests”), improved relationships (seven items, e.g., “I have a greater sense of closeness with others”), spiritual growth (two items, e.g., “I have stronger religious faith”), and appreciation of life (three items, e.g., “I changed my priorities about what is important in life”). Participants rated each item from 0 (I did not experience this as a result of my crisis) to 5 (I experienced this change to a very great degree as a result of my crisis) [23]. Full scale scores range from 0 to 105, with higher scores indicating a more positive transformation; subscales are computed with the items comprising each PTG domain. Cronbach’s alpha for the full scale was 0.95, ranging from 0.73 to 0.87 for subscales. The PTGI has both been validated in Spanish and utilized in a Puerto Rican context [46,47]. For the purposes of our study, the PTGI was administered to participants in Spanish.

#### 2.1.3. Disaster exposure

Disaster-related exposures were measured through dichotomous items derived from previous disaster mental health research. [48,49]. Participants indicated whether or not they had experienced five exposures related to Maria and Irma: physical injury directly related to the hurricanes; impact on personal health; impact on family members’ health; damage to home or appliances; and financial impact. Items were summed for a total disaster exposure score, ranging from 0 to 5, with higher scores indicating more severe exposure.

#### 2.1.4. Covariates

Covariates included age, gender, annual income, marital status, education level, social support, and geographic location. Age and income were assessed continuously, while gender and marital status were coded as binary variables (0=Male and 1=Female; 0=Unmarried and 1=Married). Educational level was coded as a four-level variable: no college attendance, some college completion, bachelor’s degree, and postgraduate work/degree. Geographical location was broken up into San Juan, the six remaining towns that make up the downtown area of Puerto Rico (i.e., metro area), and locations anywhere on the island outside the San Juan metropolitan area. All but two participants (0.4 %) identified as Hispanic/Latinx, therefore ethnicity was excluded as a covariate.

In addition, given robust research linking higher post-disaster social support to lower PTS and higher PTG [50], we included a measure of social cohesion and trust; the Social Cohesion and Trust (SCT) subscale of Sampson, Raudenbush, and Earls’ Collective Efficacy Scale (1997). Consisting of five Likert-scale rated items, participants indicated their level of agreement with three positively-framed items (e.g., “People in this neighborhood can be trusted”) and two negatively-framed and reverse coded items (e.g., “People in this neighborhood generally don’t get on with each other”) [51,52]. Cronbach’s alpha for this sample was 0.86.

### 2.2. Data analysis

Data analysis consisted of three steps. First, descriptive statistics, including percentages for categorical measures and means and standard deviations for continuous measures, were computed to provide an overview of the sample characteristics, and a correlation matrix was examined to assess bivariate relationships among study variables. Second, we examined the associations between a sum of disaster-related exposures and PTS, PTG full scale, and PTG subscales using multivariable linear regression to control for potential confounders and determine the independent effects of disaster-related exposures on these outcomes. Third, after assessing multicollinearity, we examined associations between the five specific disaster-related exposures and PTS, PTG, and PTG domains, controlling for all covariates described previously using separate multivariable linear regression models to understand distinct associations between exposures and outcomes. Data was analyzed using SAS version 9.4.

## 3. Results

### 3.1. Preliminary analyses

Table 1 presents descriptive statistics. Of the 484 participants, approximately 76 % identified as female and 55 % were unmarried, with an average age of approximately 60. In connection with Hurricanes Irma and Maria, a majority of the sample suffered financial setbacks (70 %) and witnessed damage to their homes (63 %). More than one-third of the sample dealt with health concerns among family members (37 %) as well as personally (35 %), with 5 % reporting physical injury as a result of the hurricanes. A correlation matrix with all study variables is shown in Table 2. Total disaster exposure and each specific disaster-related stressor was positively and significantly associated with PTG and PTS full scale and subscales, ranging from 0.09 to 0.35, with a few exceptions: disaster-related injury was positively, but not significantly associated with PTG, PTS, or PTG subscales, ranging from 0.05 to 0.08; disaster-induced family health effects were only associated with PTS (0.26, *p* < .001); disaster-related financial impacts were not associated with the PTG personal strength (0.06), improved relationships (0.05), and spiritual growth (0.08) subscales; and disaster-induced damage was not associated with PTG improved relationships or spiritual growth (both 0.04). Additionally, age was negatively and significantly associated with PTG (−0.12, *p* < .05), and both age and social support score were negatively and significantly associated with PTS (−0.12, *p* < .05, both), and disaster-induced financial impact (−0.18, *p* < .001 and −0.14, *p* < 0.01, respectively).

### 3.2. Multivariable models

#### 3.2.1. Full-Scale PTS and PTG

Table 3 shows the results of multivariable models with total disaster exposure predicting PTS and PTG. Table 4 summarizes the results of a model predicting PTS and another predicting PTG substituting total disaster exposure score for disaster-related stressors. Higher total disaster exposure and all five disaster-related stressors were associated with higher PTS and PTG. Full model results are available in the supplementary materials.

#### 3.2.2. PTG subscales

Table 5 presents the findings from multivariable models predicting the five PTG subscales. As shown, personal health was the most consistent unique predictor for each of the subscale items, whereas other disaster-related stressors predicted some subscales and not others. While no relationships were negative, specifically, home damage was associated with new possibilities. Financial impact was associated with the new possibilities and improved relationships subscales. Experiencing an injury was associated with all subscales but spiritual growth. However, these results were not considered statistically significant. Full model results are available in the supplementary materials.

## 4. Discussion

The current study examined relationships between exposure to Hurricanes Irma and Maria with PTS and PTG among a sample of 484 adults in Puerto Rico post-disasters. In multivariable models, we found that two different disaster-related stressors (impact on personal health and impact on family health) were each significantly associated with higher levels of both PTS and PTG. These findings are consistent with prior literature reporting both positive and adverse mental health traumatic events [53,54], and finding that higher disaster-related exposure is associated with higher levels of PTS and PTG [55]. While limited, the finding that personal and familial health presented the strongest association with PTG aligns with findings from extant research [56]. Additionally, the authors hypothesize that this association is linked to the tangible and impressionable nature of injury to oneself or those around them. The impacts of physical injury on psychological well-being are well studied, with a subset revealing that injuries – especially those more severe – force individuals to reconfigure how they go about and perceive daily life [57,58]. Injury to family members can result in a similar shift, with loved ones potentially assuming the responsibility of their care and making adjustments in their own lives in order to support family members in ways they did not prior to the disaster [59].

Among the primary findings of the study were that PTS and PTG full scale scores were elevated in individuals who were physically injured and had their overall health negatively impacted by the hurricanes. This is consistent with extant literature linking disaster-related impacts on personal health to poor mental health and wellbeing [23,60]. The results of this study, however, indicate that being physically injured and/or having health impacted by the hurricanes was associated with heightened PTS and PTG, including higher levels of the PTG domains personal strength, appreciation of life, strengthened relationships, and belief in new possibilities as a result of the hurricanes. Such findings suggest the potential to harness these domains of PTG in interventions for disaster survivors who have experienced health impacts. These findings carry some important implications. Not only do these insights identify communities of high need postdisaster, but they also pinpoint specific types of disaster exposures that might be more likely to yield different PTG domains and can therefore be leveraged to further improve mental health outcomes among survivors. For example, previous research has shown that greater PTG and decreased PTSD symptoms occur after certain types of therapies, such as Cognitive–Behavioral Conjoint and exposure therapies [61,62]. Clinical integration of PTG can also take the form of different evidence-based strategies deployed by mental health providers in their interactions with patients, such as focusing on the struggle as opposed to the event, engaging in effective listening, and serving as the “expert companion” [63]. These evidence-based interventions can be modified to target some of the most common disaster exposures amenable to increases in PTG as identified in this study, such as how disaster exposure has impacted personal health as well as that of family members. By leveraging the potential for PTG among survivors who have endured specific exposures, clinicians can tailor their interventions to maximize resilience in disaster survivors. [64–66]. It has also been shown that individuals with elevated levels of PTS may also experience higher PTG, suggesting that as PTS symptoms increase, there is more room for PTG [24]. Focusing on facilitating PTG in a clinical setting can improve PTS symptoms and foster resilience during future crises.

The hurricanes also caused significant harm to families, contributing to elevated PTS and PTG. These findings reinforce that direct impacts to loved ones are associated with adverse mental health consequences, and these experiences can also lead to aspects of PTG as an individual responds to and copes with the traumatization of those closest to them [66,67]. The death of loved ones has been a long-cited source of psychological vulnerabilities, including increased risk of PTS [66,68]; however, a more novel finding is the way in which PTG, most prominently in the form of new possibilities and appreciation of life, could occur as a result of a wider range of family impacts during and after a disaster. Further research should explore and identify pathways leading from such exposures to these PTG domains.

While research examining the linkages between socioeconomic status and post-disaster health and well-being is ample, our analysis uncovered a unique aspect of this relationship, indicating that financial harm induced by the hurricanes was associated with both PTS, PTG, and three PTG domains - new possibilities, improved relationships, and appreciation of life [69,70]. Interestingly, age and social support had negative correlations with PTG and PTS, though these findings only held for age in multivariable models. This may indicate that being younger and lacking social support may contribute to worse outcomes following a disaster, a phenomenon found by Sharp and colleagues (2018) while analyzing cancer-related financial stress and PTG [71]. Although future research is needed, these results may indicate that when finances are restricted, individuals are required to decide how to spend their funds more strategically, resulting in transformed perceptions of the power of finances in dictating an individual’s growth and future trajectory. An alternative possibility is that PTG and its domains could result as survivors draw on internal and external resources to offset financial losses and reestablish financial well-being

Lastly, the analysis revealed that only the accumulation of disaster-related stressors was associated with higher spiritual growth. One possible explanation for this pattern of results is the predominance of religiosity in Puerto Rico, where only eight percent of the population identifies as atheist, agnostic, or of no particular religion, coupled with the predominance of religion in the island’s cultural and psychosocial landscape [72,73]. As such, there might have been less of an opportunity for spiritual growth in this population, as compared to a less religious population. Additionally, only two items on the PTGI assess spiritual change, limiting the variance in this subscale and power to detect statistically significant effects. Researchers should therefore consider developing expanded inventories of spiritual change in future studies.

The statistical significance of sociodemographics within the sample with PTS and PTG also provide critical insight into the directionality of future research. For example, there was a negative association between income and PTS score as well as a positive correlation between social support and PTG. Researchers should delve deeper into these associations, identifying potential drivers behind the deteriorating effect of income and ameliorative effect of social support on mental health outcomes in the post-disaster context.

This study has several limitations. First, the sample of high-risk adults means that the findings might not generalize to the full population of Puerto Rico. Additionally, the study did not include Puerto Ricans who relocated off the island after the hurricanes, therefore potentially misrepresenting the experiences of those whose lives were most severely impacted by the storms [11,74]. Third, disaster-related stressors were assessed as binary variables, preventing us from capturing the magnitude of each impact. To address this limitation, future research could use inventories that assess the severity of each stressor. Additionally, The cross-sectional nature of the data means that we are limited in our interpretations regarding the direction of effects, although the temporal ordering of our analyses was justified given the timeframe of different measures. However, it is possible that there are bidirectional relationships between study variables. For example, PTS could have been a marker for preexisting mental health difficulties, which could have increased risk for hurricane-related exposures. Finally, the rough nature of the items used to assess disaster-related exposures is an additional limitation. Although the single items for each exposure were derived from prior research and minimized participant burden, they did not capture variation in exposure severity. For example, the association between hurricane-related personal injury and PTS or PTG is likely to differ among disaster survivors who experienced mild versus life-threatening physical injuries. Other hurricane-related exposures that have been previously linked to PTS and PTG were also not included, such as loss of a pet, bereavement, and perceived life threat [29,75,76].

These findings accentuate a need for future research into PTS and PTG and its domains following weather-related disasters within a Puerto Rican context. Additionally, they support the need for continued mental health services addressing PTS and fostering different aspects of PTG following disasters such as Maria and Irma, both within and outside of Puerto Rico. Finally, further studies should separately examine different disaster-related stressors and their association with various short- and long-term post-disaster mental health outcomes. Such research will be critical to developing a nuanced understanding of how health and well-being can be maximized in the 21st century disaster context, especially as the prevalence of environmentally-attributed mental health conditions such as eco-anxiety and solastalgia continue to mount [77,78]. The phenomenon of PTG provides a unique opportunity to help disrupt this process, as the aftermath of climate-related disasters has been a cited source of personal growth and stronger community bonds [79]. As scientists predict an increase in both the volume and virulence of hurricanes and other weather-related disasters, we must prioritize innovative and evidence-based strategies that protect and promote mental health in a changing climate.

## Supplementary Material

1

## Figures and Tables

**Table 1:** Description of the sample (*N* = 484).

Characteristic	N(%)
Age (mean ± SD)	59.8 ± 7.1
Female	367 (75.8 %)
*Yes*	117 (24.2 %
*No*	
Married	219 (45.3 %)
*Yes*	265 (54.8 %)
*No*	
Education	190 (39.3 %)
*No college*	117 (24.2 %)
*Some college*	111 (23.0 %)
*Bachelor’s*	66 (13.6 %)
*Post-graduate*	
Income (mean ± SD)	28k ± 21k
Geographic Location	145 (30.0 %)
*San Juan*	114 (23.5 %)
*Metro Area*	225 (46.5 %)
*Other*	
Disaster Impact: Financial	344 (71.1 %)
*Yes*	140 (28.9 %)
*No*	
Disaster Impact: Physical Injury	24 (5.0 %)
*Yes*	460 (95 %)
*No*	
Disaster Impact: Infrastructural Damage	307 (63.4 %)
*Yes*	177 (36.6 %)
*No*	
Disaster Impact: Personal Health	168 (34.7 %)
*Yes*	316 (65.3 %)
*No*	
Disaster Impact: Family Health	177 (36.6 %)
*Yes*	307 (63.4 %)
*No*	
Social Score (mean ± SD)	17.1 ± 4.6
Health Conditions (mean ± SD)	2.8 ± 1.9
Disaster Score (mean ± SD)	2.1 ± 1.3
PTS Score (mean ± SD)	11.1 ± 12.0
PTG Score (mean ± SD)	66.3 ± 24.8

Note: Percentages may not sum due to rounding.

**Table 2:** Correlation between all study variables (*N* = 484).

	1	2	3	4	5	6	7	8	9	10	11	12	13	14	15	16	17	18	19	20	21	22	23	24
**1. Age**	1.00																							
**2. Female**	−0.02	1.00																						
**3. Married**	0.09	−0.11*	1.00																					
**4. Some college**	−0.09*	0.01	−0.03	1.00																				
**5. Bachelor’s degree**	−0.04	0.02	−0.02	−0.31***	1.00																			
**6. Post-grad**	−0.03	0.00	−0.05	−0.23***	−0.22***	1.00																		
**7. Income**	0.00	−0.09*	0.10*	0.00	−0.01	0.05	1.00																	
**8. San Juan**	0.11*	−0.06	0.19***	−0.10*	−0.08	−0.07	0.02	1.00																
**9. Metropolitan area**	−0.07	−0.01	0.03	0.11*	0.00	0.12	0.05*	−0.52***	1.00															
**10. Social score**	0.07	−0.05	0.13*	−0.05	0.09*	0.08	0.08	0.09	−0.01	1.00														
**11. Disease count**	0.25***	0.08	−0.01	−0.07	−0.07	−0.05	−0.06	0.34***	−0.13**	0.02	1.00													
**12. Disaster score**	−0.07	0.09	−0.04	−0.01	0.03	0.09	−0.06	0.05	−0.04	−0.10*	0.18***	1.00												
**13. Disaster injury**	0.05	0.02	0.02	−0.02	−0.04	0.10	−0.01*	0.06	−0.04	−0.03	0.03	0.31***	1.00											
**14. Disaster damage**	0.00	0.01	−0.04	−0.04	0.01	0.04	−0.04	0.05	−0.01	−0.02	0.14**	0.62***	0.10*	1.00										
**15. Disaster family health**	0.00	0.07	−0.04	−0.04	−0.02	1.00	−0.06*	0.06	−0.04	−0.04	0.14**	0.64***	0.06	0.12*	1.00									
**16. Disaster personal health**	−0.04	0.11*	−0.05	−0.01	−0.01	0.02	−0.09*	0.09	−0.10*	−0.07	0.20***	0.73***	0.19***	0.27***	0.39***	1.00								
**17. Disaster financial impact**	−0.18***	0.05	0.01	0.07	0.12**	0.04	0.02	−0.08	0.06	−0.14**	−0.02	0.62***	0.04	0.26***	0.22***	0.26***	1.00							
**18. PTG score**	−0.12*	0.09*	−0.02	0.07	−0.03	−0.05	0.01	−0.03	−0.03	0.12*	−0.05	0.17***	0.07	0.09*	0.08	0.16***	0.11*	1.00						
**19. Personal strength**	−0.11*	1.00*	−0.06	0.03	−0.01	−0.04	0.01	−0.03	−0.04	0.09*	−0.06	0.15**	0.06	0.10*	0.07	0.16***	0.06	0.88***	1.00					
**20. New possibilities**	−0.15**	0.06	−0.05	0.05	−0.02	−0.05	0.01	−0.05	−0.02	0.07	−0.09	0.17***	0.05	0.10*	0.08	0.13**	0.13**	0.92***	0.76***	1.00				
**21. Improved relationships**	−0.06	0.08	0.03	0.07	−0.06	−0.04	0.01	−0.01	−0.04	0.20***	−0.04	0.11*	0.07	0.04	0.05	0.12*	0.05	0.93***	0.77***	0.80***	1.00			
**22. Spiritual growth**	−0.09	0.10*	0.00	0.08	−0.03	−0.07	0.01	−0.03	0.00	−0.01	−0.04	0.11*	0.05	0.04	0.05	0.13*	0.08	0.81***	0.64***	0.70***	0.72***	1.00		
**23. Appreciation for life**	−0.16**	0.10*	−0.01	0.06	0.01	−0.05	−0.01	0.01	−0.03	0.07	0.00	0.27***	0.08	0.15**	0.15**	0.22***	0.18***	0.85***	0.71***	0.75***	0.70***	0.67***	1.00	
**24. PTSD score**	−0.12*	0.15**	−0.09*	0.03	0.02	−0.08	−0.07	0.00	−0.09*	−0.12*	0.11*	0.35***	0.08	0.15**	0.26***	0.33***	0.17***	0.29***	0.21***	0.28***	0.22***	0.30***	0.33***	1.00

* *p* <.05,

** *p* <.01,

*** *p* <.001.

**Table 3:** Linear regressions associated with PTS and PTG score (*N* = 484).

Characteristics	PTS *β (SE)*	PTG *β (SE)*
**Age**	−0.18* (0.07)	−0.37* (0.16)
**Female**	2.75* (1.17)	4.68 (2.56)
**Married**	−1.02 (1.05)	−0.75 (2.30)
**Education**		
Some college education	−0.16 (1.31)	0.47 (2.86)
Bachelor’s degree	−0.43 (1.34)	−5.41 (2.93)
Post-graduate	−3.68* (1.62)	−8.08* (3.54)
**Income**	−0.03 (0.06)	0.07 (0.14)
**Geographic Location**		
San Juan	−1.54 (1.29)	−2.53 (2.81)
Metropolitan Area	−2.49 (1.40)	−2.75 (3.06)
**Social Score**	−0.13 (0.11)	1.00*** (0.25)
**Health Conditions**	0.50 (0.30)	−0.92 (0.65)
**Disaster Score**	2.91*** (0.40)	3.87*** (0.87)

Note: Models were run separately for PTS and PTG.

* *p* <.05,

** *p* <.01,

*** *p* <.001.

**Table 4:** Specific disaster exposures associated with PTS & PTG (*N* = 484).

Characteristics	PTS *β (SE)*	PTG *β (SE)*
**Disaster Financial Impact**	1.24 (1.23)	3.93 (2.69)
**Disaster Injury**	2.25 (2.15)	7.10 (5.17)
**Disaster Damage**	1.61 (1.48)	3.12 (2.42)
**Disaster Personal Health**	5.44*** (1.22)	6.81* (2.69)
**Disaster Family Health**	3.75*** (1.14)	1.84* (2.50)

Note: All models control for all covariates.

Note: Models were run separately for PTS and PTG and disaster exposure.

* *p* <.05,

** *p* <.01,

*** *p* <.001.

**Table 5:** Linear regressions associated with PTG subscales (*N* = 484).

Characteristics	Personal strength *β (SE)*	New possibilities *β (SE)*	Improved relationships *β (SE)*	Spiritual growth *β (SE)*	Appreciation for Life *β (SE)*
**Disaster financial impact**	0.19 (0.53)	1.40 (0.76)	1.20 (0.93)	0.45 (0.38)	0.82 (0.44)
**Disaster Injury**	1.15 (1.01)	1.65 (0.44)	2.66 (0.78)	0.62 (0.73)	1.01 (0.84)
**Disaster Damage**	0.82 (0.47)	1.15 (0.68)	0.27 (0.83)	0.07 (0.34)	0.83* (0.39)
**Disaster Personal Health**	1.48** (0.52)	1.23 (0.71)	1.94 (0.93)	0.89* (0.38)	1.14** (0.43)
**Disaster Family Health**	0.18 (0.40)	0.75 (0.71)	0.43 (0.87)	0.04 (0.35)	0.67 (0.41)

Note: All models control for all covariates.

Note: Models were run separately for each subscale and disaster exposure.

* *p* <.05,

** *p* <.01,

*** *p* < .001.
