# Supplementary material for: Beyond the storms: Exploring predictors of posttraumatic stress and posttraumatic growth among survivors of hurricanes Irma and Maria in Puerto Rico
Source: J Clim Chang Health. 2024 Nov 29;21:100365. doi: 10.1016/j.joclim.2024.100365 (PMC11870674; doi:10.1016/j.joclim.2024.100365)
Supplement: Supplementary file 1 [file mmc1.pdf]

Supplementary Table 1: Full model specific disaster exposures associated with PTS & PTG (N = 484)

	PTS	PTG
**CHARACTERISTICS**	*β (SE)*	*β (SE)*
**Disaster Financial Impact**	1.24 (1.23)	3.93 (2.69)
Age	-0.21** (0.08)	-0.38* (0.16)
Female	3.19** (1.22)	5.18* (2.59)
Married	-0.21 (1.14)	-1.17 (2.33)
Education		
Some college education	0.08 (1.39)	0.07 (2.92)
Bachelor's degree	-0.02 (1.43)	-5.68 (3.01)
Post-graduate	-1.10 (1.74)	-7.01 (3.58)
Income	-0.05 (0.10)	0.03 (0.14)
Geographic Location		
San Juan	-1.50 (1.35)	-2.11 (2.85)
Metropolitan Area	-2.80 (1.46)	-3.15 (3.01)
Social Score	-0.17 (0.11)	0.98*** (0.24)
Health Conditions	0.82** (0.31)	-0.51 (0.65)
**Disaster Injury**	2.25 (2.15)	7.10 (5.17)
Age	-0.26*** (0.08)	-0.50** (0.64)
Female	3.25** (1.23)	5.31* (2.58)
Married	0.05 (1.14)	1.94 (2.39)
Education		
Some college education	0.59 (1.39)	1.64 (2.91)
Bachelor's degree	0.76 (1.41)	-3.53 (2.97)
Post-graduate	-0.81 (1.75)	-2.76 (3.66)
Income	0.06** (0.01)	0.04 (0.14)
Geographic Location		
San Juan	-1.82 (1.35)	-2.87 (2.84)
Metropolitan Area	-2.70 (1.47)	-2.62 (3.09)
Social Score	-0.21 (0.12)	0.92*** (0.25)
Health Conditions	0.88** (0.31)	-0.57 (0.64)
**Disaster Damage**	1.61 (1.48)	3.12 (2.42)
Age	-0.24** (0.08)	-0.43** (0.16)
Female	3.34** (1.22)	5.49* (2.60)
Married	0.20 (1.13)	-0.44 (2.34)
Education		
Some college education	0.72 (1.38)	1.40 (2.91)
Bachelor's degree	0.66 (1.41)	-4.22 (2.97)
Post-graduate	-0.69 (1.73)	-6.09 (3.57)
Income	-0.05** (0.02)	0.05 (0.14)

	PTS	PTG
**CHARACTERISTICS**	*β (SE)*	*β (SE)*
Geographic Location		
San Juan	-1.81 (1.34)	-2.65 (2.86)
Metropolitan Area	-2.87* (1.47)	-3.49 (3.11)
Social Score	-0.21 (0.12)	0.88*** (0.25)
Health Conditions	0.76* (0.31)	-0.55 (0.65)
**Disaster Personal Health**	5.44*** (1.22)	6.81* (2.69)
Age	-0.20** (0.08)	-0.39* (0.16)
Female	2.59* (1.19)	4.53 (2.58)
Married	-0.01 (1.09)	-0.57 (2.31)
Education		
Some college education	0.39 (1.33)	0.88 (2.87)
Bachelor's degree	0.58 (1.36)	-4.51 (2.93)
Post-graduate	-1.38 (1.68)	-6.67 (3.53)
Income	-0.04* (0.06)	0.08 (0.14)
Geographic Location		
San Juan	-1.76 (1.29)	-2.55 (2.83)
Metropolitan Area	-2.16 (1.42)	-2.44 (3.08)
Social Score	-0.18 (0.11)	0.93*** (0.25)
Health Conditions	0.54 (0.30)	-0.85 (0.65)
**Disaster Family Health**	3.75*** (1.14)	1.84* (2.50)
Age	-0.24** (0.08)	-0.39* (0.16)
Female	2.96** (1.20)	4.53 (2.58)
Married	0.12 (1.11)	-0.57 (2.31)
Education		
Some college education	0.59 (1.35)	0.88 (2.87)
Bachelor's degree	0.64 (0.38)	-4.51 (2.93)
Post-graduate	-1.52 (1.71)	-6.67 (3.53)
Income	-0.05** (0.01)	0.08 (0.14)
Geographic Location		
San Juan	-1.80 (1.32)	-2.55 (2.83)
Metropolitan Area	-2.64 (1.44)	-2.44 (3.08)
Social Score	-0.19 (0.12)	0.93*** (0.25)
Health Conditions	0.68* (0.30)	-0.85 (0.65)

Note: Models were run separately for PTS and PTG and disaster exposure
$*p < .05$, $**p < .01$, $***p < .001$

Supplementary Table 2: Full model linear regressions associated with PTG subscales (N = 484)

CHARACTERISTICS	Personal Strength<br>*β (SE)*	New Possibilities<br>*β (SE)*	Improved Relationships<br>*β (SE)*	Spiritual Growth<br>*β (SE)*	Appreciation for Life<br>*β (SE)*
**Disaster Financial Impact**	0.19 (0.53)	1.40 (0.76)	1.20 (0.93)	0.45 (0.38)	0.82 (0.44)
Age	-0.07* (0.03)	-0.12** (0.05)	-0.06 (0.06)	-0.03 (0.02)	-0.08** (0.03)
Female	0.93 (0.51)	0.86 (0.73)	1.59 (0.89)	0.84* (0.36)	0.89* (0.42)
Married	-0.65 (0.46)	-0.67 (0.66)	0.17 (0.80)	0.03 (0.33)	-0.05 (0.38)
Education					
Some college edu.	-0.07 (0.58)	-0.28 (0.82)	-0.01 (1.01)	0.13 (0.41)	0.16 (0.48)
Bachelor's degree	-0.68 (0.60)	-1.47 (0.84)	-2.66 (1.03)	-0.64 (0.42)	-0.34 (0.49)
Post-graduate	-1.02 (0.71)	-1.92 (1.00)	-2.40 (1.23)	-0.98 (0.50)	-0.84 (0.58)
Income	0.01 (0.03)	-0.01 (0.04)	0.01 (0.04)	0.01 (0.02)	-0.01 (0.02)
Geographic Location					
San Juan	-0.43 (0.57)	-0.70 (0.80)	-0.95 (0.98)	-0.11 (0.40)	0.07 (0.47)
Metropolitan Area	-0.71 (0.61)	-0.86 (0.87)	-1.22 (1.06)	-0.12 (0.43)	-0.23 (0.51)
Social Score	0.15** (0.05)	0.21** (0.07)	0.47*** (0.09)	0.25 (0.04)	0.10* (0.04)
Health Conditions	-0.09 (0.13)	-0.22 (0.18)	-0.17 (0.87)	-0.07 (0.09)	0.05 (0.11)
**Disaster Injury**	1.15 (1.01)	1.65 (0.44)	2.66 (0.78)	0.62 (0.73)	1.01 (0.84)
Age	-0.08* (0.03)	-0.14** (0.05)	-0.09 (0.06)	-0.03 (0.02)	-0.10*** (0.03)
Female	0.94 (0.51)	0.81 (0.73)	1.62 (0.89)	0.84* (0.36)	0.91* (0.43)
Married	-0.61 (0.46)	-0.52 (0.66)	0.26 (0.80)	0.03 (0.33)	0.03 (0.38)
Education					
Some college edu.	0.05 (0.57)	0.03 (0.82)	0.27 (1.00)	0.13 (0.41)	0.37 (0.48)
Bachelor's degree	-0.50 (0.59)	-1.06 (0.83)	-2.23 (1.02)	-0.64 (0.42)	-0.02 (0.49)
Post-graduate	-1.01 (0.71)	-1.82 (1.01)	-2.33 (1.22)	-0.98 (0.50)	-0.72 (0.59)
Income	0.01 (0.03)	0.01 (0.04)	0.01 (0.05)	0.01 (0.02)	-0.01 (0.02)
Geographic Location					
San Juan	-0.52 (0.57)	-0.82 (0.80)	-1.13 (0.98)	-0.11 (0.40)	-0.06 (0.47)
Metropolitan Area	-0.72 (0.61)	-0.82 (0.87)	-1.22 (1.06)	-0.12 (0.43)	-0.24 (0.51)
Social Score	0.14** (0.05)	0.19** (0.07)	0.45*** (0.09)	0.02 (0.04)	0.08* (0.04)
Health Conditions	-0.07 (0.13)	-0.23 (0.18)	-0.13 (0.22)	-0.07 (0.09)	0.09 (0.11)
**Disaster Damage**	0.82 (0.47)	1.15 (0.68)	0.27 (0.83)	0.07 (0.34)	0.83* (0.39)
Age	-0.08* (0.03)	-0.14** (0.05)	-0.08 (0.06)	-0.03 (0.02)	-0.10*** (0.03)
Female	0.99 (0.51)	0.95 (0.73)	1.68 (0.90)	0.86* (0.37)	0.95* (0.42)
Married	-0.53 (0.46)	-0.46 (0.66)	0.34 (0.81)	0.09 (0.33)	0.11 (0.38)
Education					
Some college edu.	0.12 (0.57)	0.12 (0.82)	0.35 (1.00)	0.24 (0.41)	0.43 (0.47)
Bachelor's degree	-0.50 (0.59)	-1.02 (0.83)	-2.24* (1.02)	-0.51 (0.42)	-0.03 (0.49)
Post-graduate	-0.92 (0.70)	-1.65 (1.00)	-2.11 (1.23)	-0.89 (0.50)	-0.66 (0.58)

CHARACTERISTICS	Personal Strength<br>*β (SE)*	New Possibilities<br>*β (SE)*	Improved Relationships<br>*β (SE)*	Spiritual Growth<br>*β (SE)*	Appreciation for Life<br>*β (SE)*
Income	0.02 (0.03)	0.01 (0.04)	0.01 (0.05)	0.01 (0.02)	0.01 (0.02)
Geographic Location					
San Juan	-0.52 (0.56)	-0.86 (0.80)	-1.09 (0.98)	-0.15 (0.40)	-0.06 (0.46)
Metropolitan Area	-0.80 (0.61)	-0.95 (0.87)	-1.31 (1.07)	-0.15 (0.44)	-0.30 (0.51)
Social Score	0.14** (0.05)	0.18** (0.07)	0.45*** (0.09)	0.02 (0.03)	0.08* (0.04)
Health Conditions	-0.11 (0.13)	-0.23 (0.18)	-0.17 (0.22)	-0.07 (0.09)	0.04 (0.11)
**Disaster Personal Health**	1.48** (0.52)	1.23 (0.71)	1.94 (0.93)	0.89* (0.38)	1.14** (0.43)
Age	-0.07* (0.03)	-0.13** (0.05)	-0.07 (0.06)	-0.03 (0.02)	-0.09*** (-.-3)
Female	0.80 (0.51)	0.71 (0.73)	1.42 (0.89)	0.77* (0.36)	0.77 (0.42)
Married	-0.56 (0.46)	-0.50 (0.65)	0.33 (0.79)	0.08 (0.33)	0.07 (0.38)
Education					
Some college edu.	0.02 (0.57)	-0.01 (0.81)	0.23 (0.99)	0.20 (0.41)	0.34 (0.47)
Bachelor's degree	-0.55 (0.58)	-1.08 (0.83)	-2.34* (1.01)	-0.54 (0.41)	-0.06 (0.48)
Post-graduate	-1.03 (0.70)	-1.75 (0.99)	-2.31 (1.21)	-0.96 (0.50)	-0.75 (0.57)
Income	0.02 (0.03)	0.02 (0.04)	0.02 (0.05)	0.01 (0.02)	0.01 (0.02)
Geographic Location					
San Juan	-0.48 (0.56)	-0.83 (0.80)	-1.06 (0.97)	-0.15 (0.40)	-0.03 (0.46)
Metropolitan Area	-0.58 (0.61)	-0.70 (0.87)	-1.03 (1.06)	-0.05 (0.43)	-0.08 (0.50)
Social Score	0.15** (0.04)	0.19** (0.07)	0.46*** (0.08)	0.02 (0.03)	0.09* (0.04)
Health Conditions	-0.16 (0.13)	-0.28 (0.18)	-0.26 (0.22)	-0.11 (0.09)	-0.01 (0.11)
**Disaster Family Health**	0.18 (0.40)	0.75 (0.71)	0.43 (0.87)	0.04 (0.35)	0.67 (0.41)
Age	-0.08* (0.03)	-0.14** (0.05)	-0.08 (0.06)	-0.03 (0.02)	-0.10*** (0.03)
Female	0.90 (0.51)	0.81 (0.73)	1.54 (0.89)	0.83* (0.37)	0.84* (0.42)
Married	-0.58 (0.46)	-0.52 (0.66)	0.31 (0.80)	0.08 (0.33)	0.06 (0.38)
Education					
Some college edu.	0.05 (0.57)	0.03 (0.82)	0.27 (1.00)	0.22 (0.41)	0.37 (0.47)
Bachelor's degree	-0.52 (0.59)	-1.09 (0.83)	-2.30* (1.01)	-0.52 (0.42)	-0.05 (0.48)
Post-graduate	-1.01 (0.71)	-1.82 (1.01)	-2.32 (1.23)	-0.95 (0.50)	-0.81 (0.58)
Income	0.02 (0.03)	0.01 (0.04)	0.01 (0.04)	0.01 (0.02)	0.01 (0.02)
Geographic Location					
San Juan	-0.49 (0.57)	-0.82 (0.80)	-1.07 (0.98)	-0.14 (0.40)	-0.03 (0.47)
Metropolitan Area	-0.70 (0.62)	-0.82 (0.87)	-1.20 (1.06)	-0.12 (0.44)	-0.19 (0.51)
Social Score	0.14** (0.05)	0.19** (0.07)	0.46*** (0.09)	0.02 (0.03)	0.09* (0.04)
Health Conditions	-0.10 (0.13)	-0.23 (0.18)	-0.18 (0.22)	-0.08 (0.09)	0.04 (0.11)

Note: Models were run separately for each subscale and disaster exposure
$*p < .05$, $**p < .01$, $***p < .001$

Supplementary Table 3: Model fit statistics

	**PTS**		**PTG**		**Personal Strength**		**New Possibilities**		**Improved Relationships**		**Spiritual Growth**		**Appreciation for Life**	
**CHARACTERISTICS**	*F stat (d.f.)*	*Adj.* R^2	*F stat (d.f.)*	*Adj.* R^2	*F stat (d.f.)*	*Adj.* R^2	*F stat (d.f.)*	*Adj.* R^2	*F stat (d.f.)*	*Adj.* R^2	*F stat (d.f.)*	*Adj.* R^2	*F stat (d.f.)*	*Adj.* R^2
**Disaster Financial Impact**	4.9 (12)	0.10	3.9 (12)	0.08	2.8 (12)	0.05	3.6 (12)	0.07	4.4 (12)	0.09	2.2 (12)	0.03	3.6 (12)	0.07
**Disaster Injury**	4.5 (12)	0.09	3.7 (12)	0.07	2.9 (12)	0.05	3.2 (12)	0.06	4.5 (12)	0.09	2.1 (12)	0.03	3.0 (12)	0.05
**Disaster Damage**	4.9 (12)	0.10	3.8 (12)	0.08	3.2 (12)	0.06	3.5 (12)	0.07	4.2 (12)	0.09	2.0 (12)	0.03	3.6 (12)	0.07
**Disaster Personal Health**	7.7 (12)	0.16	4.4 (12)	0.09	3.7 (12)	0.07	3.6 (12)	0.07	4.7 (12)	0.09	2.5 (12)	0.04	4.1 (12)	0.08
**Disaster Family Health**	6.5 (12)	0.14	3.6 (12)	0.07	2.8 (12)	0.05	3.3 (12)	0.06	4.2 (12)	0.09	2.1 (12)	0.03	3.4 (12)	0.07

Note: All models control for all covariates

Note: Models were run separately for each subscale and disaster exposure
